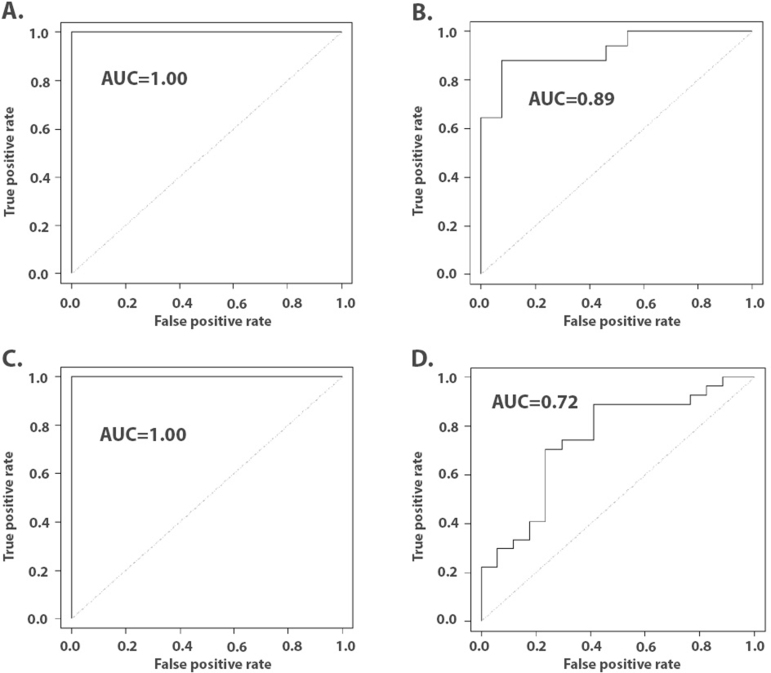# Diagnosis of early-stage non-small cell lung cancer using DNA methylation in tissue and plasma

**DOI:** 10.1016/j.gendis.2025.101548

**Published:** 2025-01-28

**Authors:** Lei Li, Kai Fu, Xuyu Cai, Dan Liu, Yingying Zhu, Weiwen Wang, Panwen Tian, Ye Wang, Hui Xue, Michael P. Snyder, Weimin Li

**Affiliations:** a Department of Respiratory and Critical Care Medicine, West China Hospital, Sichuan University, Chengdu, Sichuan 610000, China; b Genetics Department, Center for Personalized Medicine, Stanford University, CA 94303, USA; c Precision Medicine Research Center, West China Hospital, Sichuan University, Chengdu, Sichuan 610000, China; d Frontiers Science Center for Disease-related Molecular Network, Sichuan University, Chengdu, Sichuan 610000, China; e The Research Units of West China, Chinese Academy of Medical Sciences, West China Hospital, Chengdu, Sichuan 610000, China

**Keywords:** Circulating tumor DNA, Diagnosis, Liquid biopsy, Methylation, Non-small cell lung cancer

## Abstract

DNA methylation is a key epigenetic alteration in tumorigenesis, but its diagnostic value in early-stage lung cancer remains unclear. In this study, tissue and plasma samples from patients with lung cancer or benignity were analyzed. Methylation profiles were obtained using bisulfite sequencing and compared with selected lung cancer-specific markers. Diagnostic prediction models were constructed using these markers, with their efficacy assessed by sensitivity, specificity and area under the curve (AUC). In the tissue cohort, 276 markers were found to be significantly differentially methylated in lung cancer (FDR < 0.05). A diagnostic prediction model using six markers showed promising performance in both the training cohort (sensitivity = 90%; specificity = 97%; AUC = 0.988) and the validation cohort (sensitivity = 92%; specificity = 94%; AUC = 0.977). In the plasma cohort, a diagnostic prediction model using nine markers achieved a sensitivity of 98% and specificity of 100% (AUC = 0.998) in the training cohort, a sensitivity of 81% and specificity of 59% (AUC = 0.791) in the validation cohort. Furthermore, we observed a significant correlation between delta methylation changes in tissue and plasma in the paired patient cohort. Additional analysis based on methylation haplotypes identified 1222 differentially methylated regions in tissue samples, mainly enriched in DNA replication-related pathways. Additionally, correlations between DNA methylation and clinical characteristics revealed significant differential methylation patterns between smokers and non-smokers . Thus, DNA methylation in both tissue and plasma holds potential as a biomarker for the early diagnosis of lung cancer.

## Introduction

Detecting lung cancer at its early, localized stage offers the best opportunity for a complete cure and for patients to remain free from further medical interventions and complications. With the extensive application of low-dose computed tomography, it is now possible to detect lung cancer at a very early stage. However, the numerous false-positive cases have raised new issues and concerns.^1^ Various methods, including positron emission tomography/computed tomography, fibrobronchoscopy, and transthoracic needle biopsy, are employed to differentiate lung cancer from benign disease. However, the diagnostic accuracy remains unfavorable, especially in early-stage lung cancer.^2^

DNA methylation typically occurs on cytosine residues at the 5-carbon position, often around gene promoters in CpG islands.^3^ During tumorigenesis, methylation alterations have been detected at a very early stage in various tumor types and across many genomic regions. Furthermore, DNA methylation can help determine the origin of malignancy, making it an ideal approach for the early diagnosis of lung cancer.^4^ Previous studies have successfully recognized lung cancer by detecting altered methylation of genes such as SHOX2, RASSF1A, p16, and SEPT9^5^ in tissue samples. Considering the accessibility and noninvasiveness compared with tissue biopsy, methylation patterns in circulating tumor DNA (ctDNA) have garnered attention as a diagnostic tool. Released by tumor cells through necrosis, apoptosis, spontaneous secretion, and circulating tumor cells,^6^ ctDNA can be isolated from plasma, lavage, sputum, or pleural fluid in cancer patients. It can be collected at different time points, enabling real-time monitoring of molecular changes and tumor progression.^7^ With the advent of next-generation sequencing and bioinformatics, methylation alterations at numerous CpG sites are being explored and analyzed in a high-throughput manner. Its feasibility in lung cancer diagnosis has been demonstrated in previous studies,^8^^,^^9^ but the efficacy remains unclear, particularly for early-stage lung cancer.

In this study, we utilized capture-based bisulfite sequencing to detect DNA methylation status in tissue and plasma samples, simultaneously targeting approximately two hundred thousand cancer-related CpG sites. Both single CpG site-based analysis and methylation haplotype-based analysis were employed to evaluate the diagnostic performance of DNA methylation in lung cancer. Compared with previous studies, our focus was primarily on early-stage lung cancer patients (especially those with nodules smaller than 3 cm) to distinguish malignancy from benignity, addressing a critical unmet clinical need.

## Material and methods

### Clinical study design and sample collection

The non-invasive lung cancer screening project is an ongoing prospective study initiated in 2015 at the West China Medical Center, aimed at identifying biomarkers that can predict early-stage lung cancer in various clinical sample types: peripheral blood, sputum, and bronchoalveolar lavage fluid. Adults with positive computed tomography scans indicating suspicious pulmonary nodules 5 mm or greater in diameter were enrolled. Exclusion criteria included pregnant or lactating females and patients with pathologically confirmed cancers originating from other organs. Detailed clinical information, including demographics, imaging parameters (such as diameter, density, and location), and other laboratory tests, such as carcinoembryonic antigen (CEA), neuron-specific enolase (NSE), and cytokeratin 19 fragment (CYFRA21-1), were collected at enrollment. All tissue specimens were collected during surgery and preserved as formalin-fixed paraffin-embedded slices. Peripheral blood samples (10 mL) were collected 1–7 days prior to surgery using Streck-BCT tubes. Participants who received a pathologically confirmed diagnosis were included in the subsequent analysis (Table 1). The clinical protocol of this study was approved by the Ethics Committee of the West China Medical Center (2017(114)), and written informed consent was obtained from all participants in this research.

**Table 1:** The clinicopathologic features of cohorts in this study.

Patient characteristics, *n* (%)	Tissue			Plasma		
	Malignant	Benign	Total	Malignant	Benign	Total
	(*n* = 56)	(*n* = 44)	(*n* = 100)	(*n* = 89)	(*n* = 57)	(*n* = 146)
Age at diagnosis, *n* (%)						
≤40	2 (3.6)	14 (31.8)	16 (16.0)	5 (5.6)	12 (21.1)	17 (11.6)
41–55	16 (28.6)	17 (38.6)	33 (33.0)	24 (27.0)	25 (43.9)	49 (33.6)
56–70	34 (60.7)	11 (25.0)	45 (45.0)	52 (58.4)	19 (33.3)	71 (48.6)
≥71	4 (7.1)	2 (4.5)	6 (6.0)	8 (9.0)	1 (1.8)	9 (6.2)
Gender, *n* (%)						
Male	26 (46.4)	31 (70.5)	57 (57.0)	40 (44.9)	31 (54.4)	71 (48.6)
Female	30 (53.6)	13 (29.5)	43 (43.0)	49 (55.1)	26 (45.6)	75 (51.4)
Stage, *n* (%)						
I	41 (73.2)	–	–	62 (69.7)	–	–
II	5 (8.9)	–	–	8 (9.0)	–	–
III/IV	10 (17.9)	–	–	19 (21.3)	–	–
Smoking history, *n* (%)						
Former or current smoker	19 (33.9)	25 (56.8)	44 (44.0)	30 (33.7)	20 (35.1)	50 (34.2)
Never-smoker	37 (66.1)	19 (43.2)	56 (56.0)	59 (66.3)	37 (65.0)	96 (65.8)
Histology, *n* (%)						
Adenocarcinoma	48 (85.7)	–	–	73 (82.0)	–	–
Squamous cell carcinoma	8 (14.3)	–	–	9 (10.1)	–	–
Others^a^	0.0 (0.0)	–	–	7 (7.9)	–	–
Atypical adenomatous hyperplasia	–	1 (2.3)	–	–	2 (3.5)	–
Inflammation	–	11 (25.0)	–	–	24 (42.1)	–
Tuberculosis	–	25 (56.8)	–	–	17 (29.8)	–
Benign tumors^b^	–	6 (13.6)	–	–	12 (21.1)	–
Fungal infection	–	1 (2.3)	–	–	2 (3.5)	–
Nodule size, *n* (%)						
None or <0.5 cm	0 (0.0)	0 (0.0)	0 (0.0)	0 (0.0)	0 (0.0)	0 (0.0)
0.5–1 cm	5 (8.9)	6 (13.6)	11 (11.0)	6 (6.7)	15 (26.3)	21 (14.4)
1–2 cm	20 (35.7)	12 (27.3)	32 (32.0)	31 (34.8)	13 (22.8)	44 (30.1)
2–3 cm	15 (26.8)	13 (29.5)	28 (28.0)	24 (27.0)	6 (10.5)	30 (20.5)
>3 cm	16 (28.6)	10 (22.7)	26 (26.0)	28 (31.5)	14 (24.6)	42 (28.8)
Unknown^c^	0.0 (0.0)	3 (6.8)	3 (3)	0 (0.0)	9 (15.8)	9 (6.2)

Note: Others^a^ include adenosquamous carcinoma, neuroendocrine carcinoma, large cell carcinoma, and small cell carcinoma. Benign tumors^b^ include hamartoma, sclerosing hemangioma, fibrous tumor, and inflammatory myofibroblastic tumor. The unknown nodule size^c^ was not obtained because its CT image was unavailable or it was unmeasurable due to atelectasis.

### The isolation of tissue genomic DNA and plasma circulating cell-free DNA

Tissue genomic DNA (gDNA) was isolated from the formalin-fixed paraffin-embedded tissue samples using the Qiagen QIAamp DNA FFPE Tissue Kit (Qiagen, Cat# 56404) according to the manufacturer's instructions. The gDNA was then fragmented to 200 bp using the M220 Focused-ultrasonicator™ (Covaris, Inc., Massachusetts, USA) following the manufacturer's protocol. A total of 100 ng of fragmented DNA was used for library construction.

Plasma circulating cell-free DNA (cfDNA) was isolated using the Qiagen QIAamp Circulating Nucleic Acid Kit (Qiagen, Cat# 55114) according to the manufacturer's protocol. To prevent degradation and contamination from white blood cells, repeated freezing and thawing of plasma was avoided. The concentration of cfDNA was measured using the Qubit™ dsDNA HS Assay Kit (Thermo Fisher Scientific, Cat# Q32854), and its quality was assessed using the Agilent High Sensitivity DNA Kit (Cat# 5067-4626). cfDNA with a yield greater than 3 ng and without over-contamination by gDNA was used for library construction.

### Bisulfite conversion and AnchorIRIS™ targeted methylation sequencing

Bisulfite conversion was performed using the Zymo Lightning Conversion Reagent (Cat# D5031, Zymo Research) following the manufacturer's protocol.

Library preparation was completed using the AnchorIRISTM assay with the AnchorDx EpiVisioTM Methylation Library Prep Kit (AnchorDx, Cat# A0UX00019) and the AnchorDx EpiVisioTM Indexing PCR Kit (AnchorDx, Cat# A2DX00025). This assay directly ligates adaptors to the 3′ end of single-stranded DNA molecules after bisulfite conversion and can achieve a detection limit of 0.0033%. Target enrichment was carried out using the AnchorDx EpiVisioTM Target Enrichment Kit (AnchorDx, Cat# A0UX00031). A custom-made 10K methylation panel, which includes 9921 preselected regions enriched for cancer-specific methylation, was utilized in this process. Detailed information about this assay can be found in a previous study.^10^

### Sequencing data analysis pipeline

Quality control of the raw sequencing data was performed using FastQC Version 0.11.8 (https://www.bioinformatics.babraham.ac.uk/projects/fastqc/). Parameters such as per base sequence quality, per tile sequence quality, per sequence quality scores, per base sequence content, per sequence GC content, sequence length distribution, and adaptor content were evaluated. Sequencing adaptors and bases with poor quality were then trimmed using Cutadapt V2.0 (https://cutadapt.readthedocs.io/en/stable/index.html).

Subsequently, the trimmed reads were aligned to the hg19 genome using BS-Seeker2 (https://github.com/BSSeeker/BSseeker2). The methylation status was calculated by comparing the C/T value with that of the reference genome, taking into consideration the library conversion efficiency. Finally, customized R functions were employed to perform the differential methylation analysis. Only high-confidence CpG sites (>30× coverage) were selected for further data analysis.

### DNA methylation marker preselection

The samples were randomly divided into the training and validation cohorts at a ratio of 7:3. In the training cohort, the Wilcoxon rank sum test was used to compare lung cancer with benign diseases, identifying statistically significant differences. The Wilcoxon rank sum test was chosen because the methylation levels did not follow a normal distribution but rather exhibited bimodal distributions with peaks at 0 (least methylated) and 1 (fully methylated). The *p* value for each marker was adjusted for multiple testing using the Benjamini–Hochberg method to achieve a false discovery rate (FDR).

Delta methylation values were calculated to represent the average differences in methylation levels. Due to the variability of captured methylation fragments across samples, we required the identified CpG sites to be measured in at least 65% of patients. Differentially methylated CpG sites (DMSs) were identified for further analysis based on FDR, delta methylation change, and occurrence frequency in both lung cancer and benign diseases.

### Building a diagnostic model

To reduce the number of identified DMSs, we applied the least absolute shrinkage and selection operator (LASSO) machine learning method. We subsampled the data in the training cohort without replacement 500 times and selected DMSs with repeat occurrence frequencies greater than 450. The tuning parameters for LASSO were determined based on the expected generalization error estimated from 10-fold cross-validation and information-based criteria (Akaike's information criterion and Bayesian information criterion). The largest lambda value that controlled the error within one standard error of the minimum, known as the “1-se” lambda, was adopted.

The markers chosen by LASSO were then used to build the diagnostic model. For the sake of interpretability, we fitted a simple logistic regression model using these markers as covariates. In addition, we also included age, gender, and smoking status as covariates in our model to control for potential confounding factors. A combined diagnostic score was obtained by multiplying the unbiased coefficient estimates by the marker methylation value matrix in both the training and validation cohorts. The diagnostic performance of this model was evaluated by sensitivity, specificity, accuracy, and area under the receiver operating characteristic curve (AUC) when differentiating lung cancer from benign disease.

The probability score, calculated by the logistic regression model, indicated the consistency between the pathological results and the methylation model predicted results. This score ranged from 0 to 1; the closer it was to 1, the more likely the predicted results were correct. Additionally, the diagnostic performances of the plasma methylation models were compared with those of conventional tumor markers, including CEA, NSE, and CYFRA 21-1.

## Results

### Participants and sample characteristics

From September 2015 to December 2017, we enrolled 517 participants with chest CT-determined pulmonary nodules from West China Hospital (Fig. 1). A total of 100 tissue samples and 146 plasma samples from 214 participants were sequenced and analyzed, with 48 participants providing both tissue and plasma samples. The clinical characteristics of these participants are summarized in Table 1. Smoking status was the only variable that differed slightly between participants with lung cancer and those with benign diseases, while other variables (such as age, gender, and nodule size) were similar. Notably, the majority (73.2%, 41/56) of the enrolled lung cancer patients were diagnosed at stage Ⅰ, with a nodule size of less than 3 cm.

**Figure 1:**
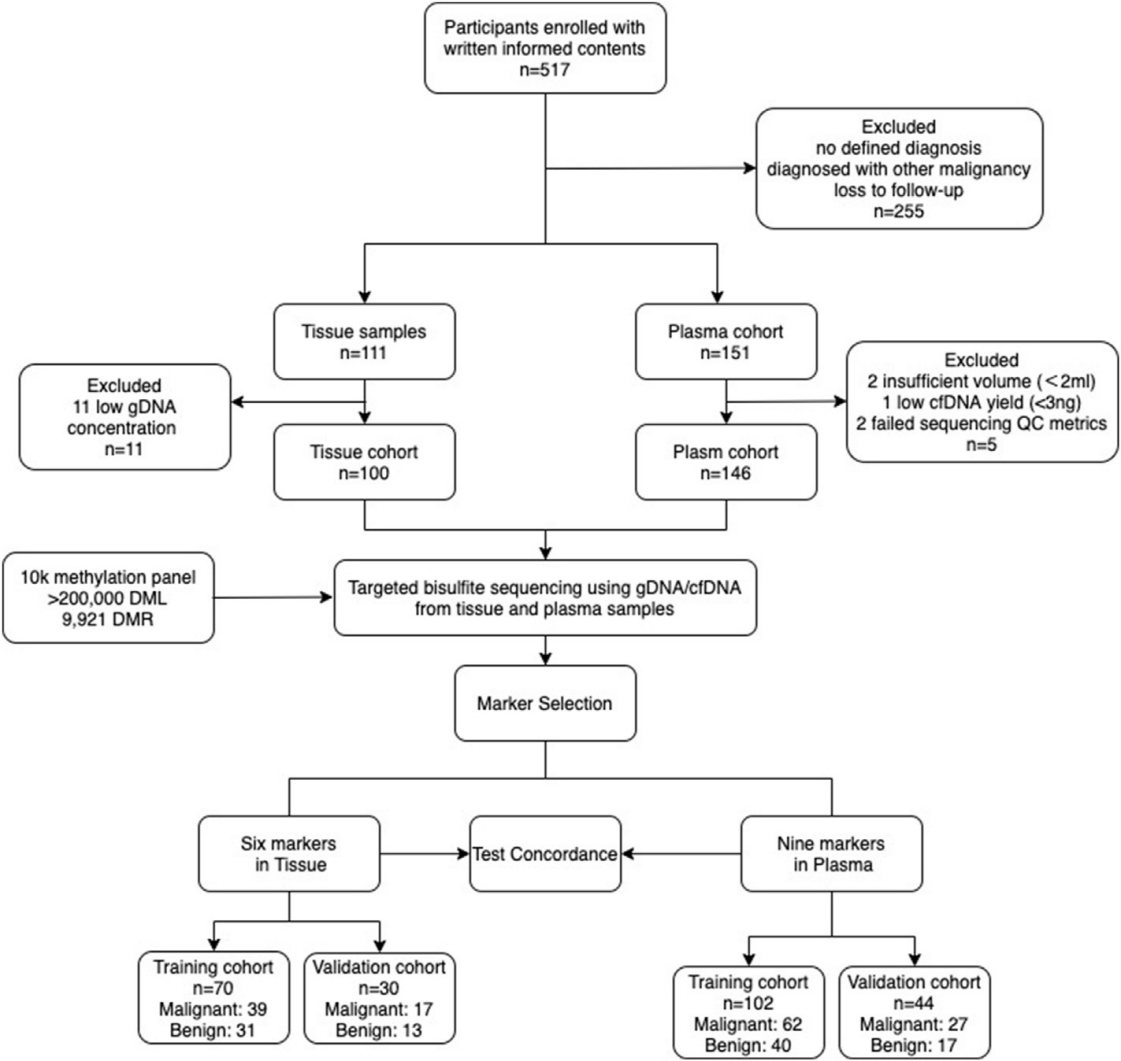
Study flow of subject enrollment and model generation. A total of 517 participants were enrolled. Tissue and plasma samples were collected, and the 10 k methylation panel was applied to access the methylation levels. Lung cancer-specific biomarkers were then selected to develop the diagnostic models, which were then validated in different samples.

### Identification of lung cancer-specific methylation markers in tissue

To identify lung cancer-specific methylation signatures, we randomly split the enrolled tissue samples into a training cohort (*n* = 70) and a validation cohort (*n* = 30) in a 7:3 ratio. In the training cohort, 40 lung cancer tissue samples and 30 benign tissue samples were analyzed. The Wilcoxon rank sum test was applied to compare the methylation status of approximately 200,000 CpG sites, followed by the Benjamini–Hochberg method to adjust the FDR. A total of 276 DMSs were identified under the cutoff criteria of delta methylation change ≥0.15 and FDR < 0.05. Additionally, all identified DMSs were required to be detected in at least 65% of samples. These sites were significantly enriched in noncoding RNA (5.6-fold), promoter (3.9-fold), and 5′ untranslated region (5′UTR) (2.1-fold) sequences. Unsupervised hierarchical clustering demonstrated that these DMSs could distinguish lung cancer from benign diseases (Fig. 2A). Notably, the methylation pattern of sclerosing hemangioma was prominently different from those of other benign diseases.

**Figure 2:**
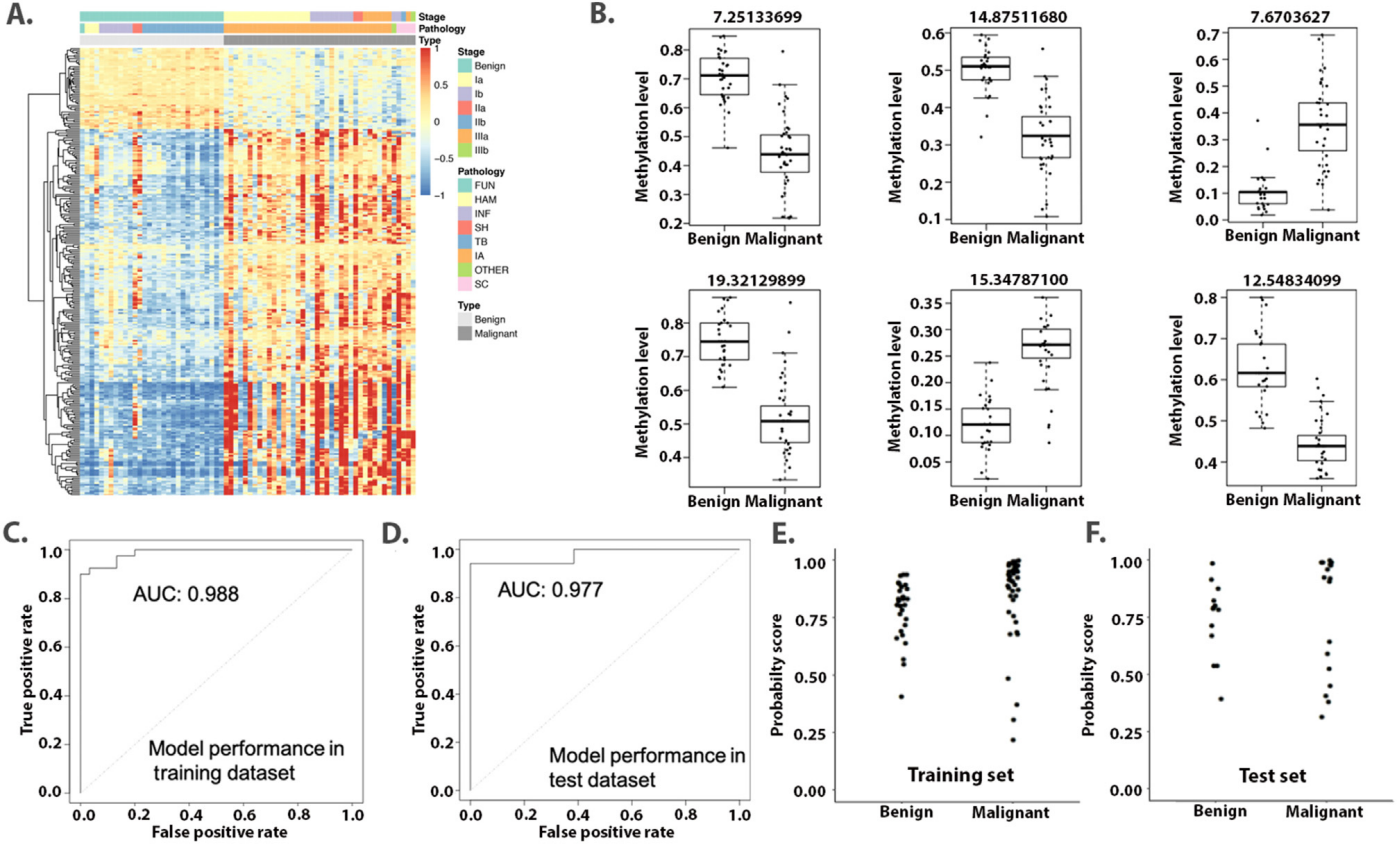
Differential methylation in the tissue cohort. **(A)** Unsupervised hierarchical clustering of 276 differential methylation sites selected in the tissue samples. **(B)** The methylation levels of 6 differential methylation sites selected for the diagnostic prediction model. **(C, D)** Receiver operator characteristic curves of the diagnostic prediction model in the training set (C) and validation set (D). **(E, F)** Distribution of probability scores (range, 0 to 1) of a diagnostic prediction model in the training set (E) and validation set (F).

We then applied the LASSO method to select the most informative methylation signatures and reduce the number of candidate methylation markers (Fig. S1A). Six out of the 276 markers were identified, showing significantly different methylation statuses between lung cancer and benign diseases (Fig. 2B). Among these six markers, two were hypermethylated in lung cancer, while four were hypermethylated in benign diseases. Their characteristics are summarized in Table S1. Further downstream analysis revealed that these differentially methylated sites were significantly enriched in pathways related to the transcriptional regulatory network in embryonic stem cells. Previous studies have found differential methylation of ZNF316^11^ and MIR1233-1^12^ in cancer cell lines. Additionally, CYCS^13^ and THEG5^14^ were differentially expressed in breast cancer and prostate cancer, while copy number variation of LOC283585^15^ was identified in colorectal cancer. No previous data were found for LOC102724050, necessitating further studies to characterize its role in tumorigenesis.

Using the logistic regression method, we then constructed a diagnostic prediction model with these six markers. The model weight of each marker is shown in Table S1. The model yielded a sensitivity of 90% (36/40) and a specificity of 97% (29/30) in the training cohort (Table 2), and a sensitivity of 92% (12/13) and a specificity of 94% (16/17) in the validation cohort. This diagnostic model successfully differentiated lung cancer from benign diseases in both the training (AUC = 0.988) (Fig. 2C) and validation cohorts (AUC = 0.977) (Fig. 2D). The efficacy of the model was further confirmed using probability scores (Fig. 2E and F).

**Table 2:** The diagnostic performances of the 6-marker prediction model in tissue and the 9-marker prediction model in plasma.

	Tissue cohort				Plasma cohort			
	Training set		Validating set		Training set		Validating set	
	Malignant	Benign	Malignant	Benign	Malignant	Benign	Malignant	Benign
Predicted malignant	36	1	12	1	61	0	22	7
Predicted benign	4	29	1	16	1	40	5	10
Total	40	30	13	17	62	40	27	17
Sensitivity (%)	90		92		98		81	
Specificity (%)	97		94		100		59	
Positive predictive value	0.973		0.923		1.00		0.759	
Negative predictive value	0.879		0.941		0.976		0.667	
Accuracy	0.929		0.933		0.990		0.727	

### Concordance of DNA methylation between tissue and plasma

To explore the concordance between tissue and plasma, we narrowed our analysis to paired tissue and plasma samples. In the paired patient cohort, we first identified DMSs between malignant and benign tissue samples and then examined their delta methylation levels in the plasma samples. We observed a significant correlation (*p* = 0.004, Spearman correlation test) between the delta methylation levels of tissue and plasma samples (Fig. S2A). This finding is consistent with previous studies showing that plasma methylation can reflect tissue methylation levels.

We also analyzed all tissue samples and examined their methylation status in plasma samples. No significant correlations were found between methylation changes in tissue and plasma samples (Fig. S2B). We further classified the 276 sites into two groups: concordance (including sites hypermethylated or hypomethylated in both tissue and plasma samples) and discordance (including sites hypermethylated in tissue samples while hypomethylated in plasma samples, and *vice versa*). As shown in Fig. S2C, most sites were in the concordance group, indicating that plasma samples also tended to have higher or lower methylation levels when tissue samples were hypermethylated or hypomethylated. Although no obvious quantitative correlations were found, there was a qualitative relationship between the different samples.

### Identification of lung cancer-specific methylation markers in plasma

We only observed DNA methylation concordance in the paired samples, which only had a limited number of sample size. Thus, we then used all the plasma samples to construct a plasma-based model. A total of 146 plasma samples were randomly split into a training cohort (*n* = 102) and a validation cohort (*n* = 44) in a 7:3 ratio. Among the 277,978 CpG sites detected in the training cohort, 147,623 sites were detected in more than 65% of the samples. The Wilcoxon rank sum test was used to compare methylation between lung cancer and benign diseases, with the Benjamini–Hochberg method applied to adjust the *p* values. No CpG sites met the threshold of FDR < 0.05; therefore, we set the cutoff at FDR < 0.2 and *p* < 0.001. In this process, 30 DMSs were selected with an average delta methylation change ≥0.02. These sites were significantly enriched in the promoter (4.8-fold), exon (3.8-fold), and 5′UTR (5.3-fold) sequences. Hierarchical clustering indicated that these DMSs could differentiate lung cancer from benign diseases (Fig. 3A).

**Figure 3:**
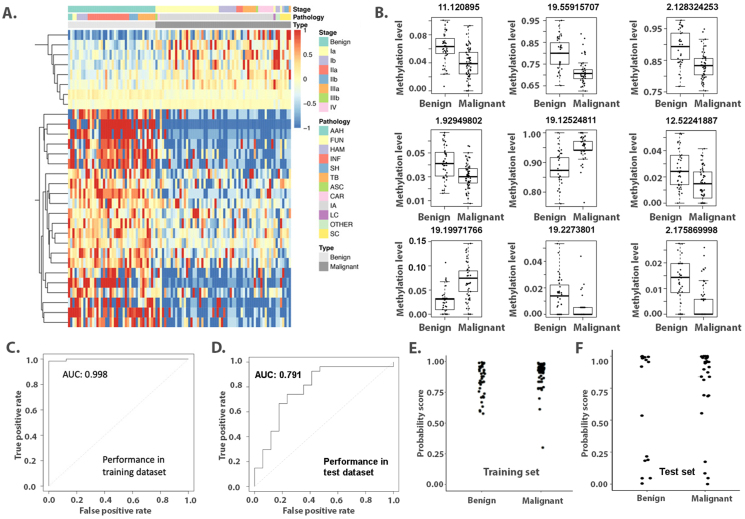
Differential methylation in the plasma cohort. **(A)** Unsupervised hierarchical clustering of 30 differential methylation sites selected in the plasma samples. **(B)** The methylation levels of 9 differential methylation sites selected for the diagnostic prediction model. **(C, D)** Receiver operator characteristic curves of the diagnostic prediction model in the training set (C) and validation set (D). **(E, F)** Distribution of probability scores (range, 0 to 1) of a diagnostic prediction model in the training set (E) and validation set (F).

LASSO regression analysis was subsequently applied, selecting the best combination of 9 CpG sites from the identified DMSs (Fig. S1C). Among these sites, only 2 were hypermethylated in lung cancer, while the remaining 7 were hypomethylated (Fig. 3B). Their characteristics are summarized in Table S2. For these genes, FIGNL2 and GFI1 have been studied in lung cancer; FIGNL2 promotes cell growth and tumorigenesis,^16^ while GFI1 shows methylation alteration.^17^ Other genes, such as TBCEL, UBE2S, and ZNF799, have been studied for their methylation changes and expression alterations, with established functions in multiple cancers.^18^ CHN1 is related to lymphoma and cervical carcinoma, though not previously studied in lung cancer.^19^ Notably, PEAK3^20^ and myo7b^21^ promote cell proliferation, migration, and invasion in *in vitro* studies. No research has been published on ZNF253.

We then constructed a diagnostic prediction model based on these 9 sites using logistic regression. The model weight of each site is shown in Table S2. The model yielded a sensitivity of 98% (61/62) and a specificity of 100% (40/40) in the training cohort (Table 2), effectively differentiating lung cancer from benign diseases (AUC = 0.998, Fig. 3C). However, the diagnostic efficacy decreased in the validation cohort (AUC = 0.791, Fig. 3D), with a sensitivity of 81% (22/27) and a specificity of 59% (10/17) (Table 2). We also calculated the probability score based on methylation patterns, which effectively identified malignancy (Fig. 3E, F). Additionally, we compared the diagnostic performances of the methylation model and conventional tumor markers extensively used in clinical practice. The methylation model significantly outperformed the conventional markers (Fig. S3). This indicates that plasma-derived methylation biomarkers can provide valuable information for early cancer diagnosis; however, the sensitivity and specificity of the plasma-based method are lower than those of the tissue-based method and require improvement in future studies.

### Methylation haplotype-based analysis in tissue and plasma

Analysis based on methylation haplotypes was conducted on both tissue and plasma samples. Methylated haplotype regions were identified according to Zhang's method^29^, considering tissue-specific methylation haplotype blocks across the genome and proposing the methylated haplotype load. In total, 20,000 of 146,888 regions with methylation signals were detected in our dataset (Table S3). With an FDR threshold of <0.05, 1222 significant DMRs were identified in the tissue samples (Table S4).

After assigning each DMR to its nearest gene, we found that DMRs frequently occurred in genes such as ULBP1, PRSS1, and EPHB6, suggesting significant methylation changes in these genes (Fig. S4A). Notably, ULBP1 is an immune system-activating receptor on natural killer cells and T cells,^22^ and EPHB6 has been shown to suppress tumor invasion and metastasis.^23^ Methylation changes in these genes may interfere with immunity and promote tumorigenesis. Further gene ontology enrichment analysis revealed that the DMRs were significantly enriched in biological processes related to DNA replication, chromatin assembly, and natural killer cell-mediated immunity (Fig. S4B). These regions were also significantly enriched in DNA replication pathways. In the plasma samples, three regions were identified with FDR < 0.2, but no regions were identified with FDR ≤ 0.05 (Table S5).

We further tried to select specific DMRs related to lung cancer diagnosis. The LASSO model was used for DMR-based marker selection in both the tissue (Fig. S5A) and plasma cohorts (Fig. S5B). The diagnostic performances are shown in Figure S5. For the tissue cohort, the AUC was 1.00 in the training set (Fig. S6A) and 0.89 in the validation set (Fig. S6B); for the plasma cohort, the AUC was 1.00 in the training set (Fig. S6C) and 0.72 in the validation set (Fig. S6D).

### Correlations between clinical characteristics and DNA methylation

Subgroup analysis was conducted based on the clinical characteristics of the patients in both the tissue and plasma cohorts. In the tissue samples, different methylation patterns were observed for smoking status (Fig. 4A), pathological type (Fig. 4B), and gender (Fig. 4C). The most prominent methylation difference was found between smokers and nonsmokers, with 172 significant DMSs (FDR ≤ 0.05) (Table S6). Compared with squamous cell carcinoma, we identified 1096 DMSs (FDR < 0.1) in adenocarcinoma (Table S6). In the gender comparison, 753 DMSs were identified, mostly located on the X chromosome as expected (Table S6). No DMSs were found between the tissue samples of older (≥55 years) and younger (<55 years) individuals (Table S6).

**Figure 4:**
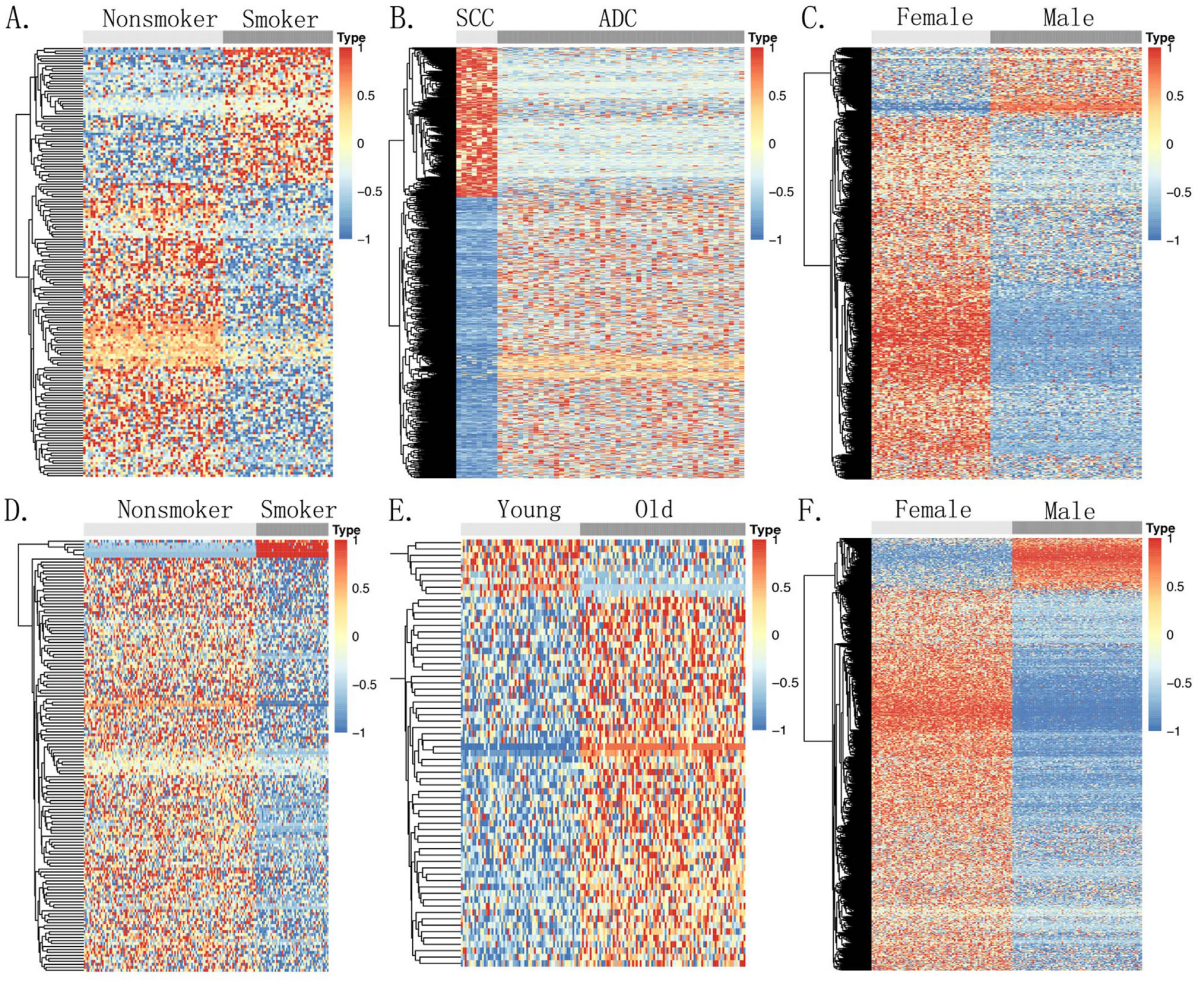
The relationships between methylation alterations and clinical characteristics. **(A)** The relationship between smoking status and methylation in tissue samples. **(B)** The relationship between pathological types and methylation in tissue samples. **(C)** The relationship between gender and methylation in tissue samples. **(D)** The relationship between smoking status and methylation in plasma samples. **(E)** The relationship between age and methylation in plasma samples. **(F)** The relationship between gender and methylation in plasma samples. SCC, squamous cell carcinoma; ADC, adenocarcinoma.

In plasma samples, cfDNA methylation was highly correlated with smoking status (Fig. 4D), with 146 DMSs (FDR < 0.05) identified between smokers and nonsmokers (Table S7). Differential methylation was also observed in the age comparison (Fig. 4E), with 67 DMSs (FDR < 0.05) between older (>55 years) and younger (≤55 years) individuals (Table S7). When compared with females (Fig. 4F), we identified 863 DMSs in males, predominantly located on the X chromosome (Table S7). No DMSs were found between different pathological types in plasma samples (Table S7).

## Discussion

Diagnosing lung cancer at an early stage is an urgent problem in clinical practice. In 2011, the National Lung Screening Trial reported that low-dose computed tomography decreased lung cancer-specific mortality by 20% compared with chest X-rays, demonstrating its superiority in lung cancer screening. However, it suffers from low specificity, with a false positive rate as high as 96.4%.^1^ Suspicious pulmonary nodules detected by low-dose computed tomography are subjected to subsequent monitoring with additional low-dose computed tomography scans or direct tissue biopsy. Since tissue remains the “gold standard” for histologic subtyping and for detection of targetable genetic alterations, invasive procedures (including fibrobronchoscopy, transthoracic needle biopsy, and surgical lung biopsy) are indispensable when malignancy is highly considered.^2^ However, they may come up against limited sensitivity, potential medical complications, and severe physical and psychological problems.

When tissue biopsy is considered to impose a great risk for the patient, liquid biopsy may work as an alternative. Substantial evidence supports the technical and clinical feasibility of detecting tumor-derived circulating nucleic acids (*e.g.*, ctDNA and ctRNA) from the plasma of cancer patients. Forms of cancer-specific somatic alterations include somatic mutations (*e.g.*, single-nucleotide variants, indels, and rearrangements), somatic copy number variations and aneuploidies, epigenetic alterations, and RNA/miRNA differential expression.^24^ The somatic mutation profiling of ctDNA has been most extensively investigated. In 2014, an assay named cancer personalized profiling by deep sequencing (CAPP-Seq) was developed, with 96% specificity for mutant allele fractions down to ∼0.02%. However, this assay achieved a sensitivity of only 50% (2/4) in stage Ⅰ lung cancer. Additionally, in one case of stage Ⅰa lung cancer, no mutation was identified.^25^ Integrated digital error suppression (iDES) was then applied to enhance CAPP-Seq, improving the detection limits to 0.004% but not achieving improvement in early-stage lung cancer.^26^ DNA methylation has the potential for substantial advantage and has attracted great attention recently.

In this research, DNA methylation analysis of tissue samples revealed differential methylation patterns in lung cancer, and a diagnostic prediction model based on these patterns could effectively differentiate lung cancer from benign diseases. This phenomenon has been demonstrated previously using data from public databases^27^^,^^28^ or newly generated sequencing data.^29^ In Hoque's study, six methylation signatures identified from The Cancer Genome Atlas (TCGA) were used to predict lung cancer, achieving a sensitivity of 92.2% and a specificity of 72.0%.^28^ Zhang et al^27^ also demonstrated comparable diagnostic performance of DNA methylation using normal lung tissue instead of benign diseases as control. In this research, we developed a 6-marker methylation model and applied benign diseases as control, achieving comparative diagnostic performances with previous studies. It further demonstrated the diagnostic value of DNA methylation in lung cancer, especially when the tissue sample is inadequate for traditional pathological diagnosis.

Based on the methylation change detected in plasma samples, we constructed a diagnostic prediction model using 9 differentially methylated markers. Multiple studies have previously explored the diagnostic value of ctDNA methylation. Initially, only a limited number of markers could be detected using low-throughput techniques. Some genes, including SHOX2, RASSF1A, SEPT9, APC, RARB, SOX17, and DAPK, were proven to have diagnostic importance, but unstable accuracies (from 7% to 84%) limited their clinical application.^30^^,^^31^ To improve diagnostic performance, multiple markers were subsequently combined in various samples (including sputum, bronchial lavage, and pleural effusion), but no significant progress was made.^32^, ^33^, ^34^

Breakthroughs have been achieved in recent years, as next-generation sequencing has been extensively applied to detect methylation alterations at numerous sites, in both gene ORFs and massive noncoding areas. In hepatocellular carcinoma, Zhang et al^35^ developed a 10-marker classifier using deep sequencing of bis-DNA target-captured with molecular-inversion (padlock) probes, achieving a sensitivity of 83.3% and a specificity of 90.5% in the validating cohort (*n* = 658). Xu et al^36^ proposed “CancerDetector” based on cfDNA methylation profiles, which also achieved outstanding diagnostic performance (sensitivity 94.9%, specificity 100%), while the sample size was limited (*n* = 49). This team further developed a 9-marker methylation classifier to help diagnose colorectal cancer, achieving a sensitivity of 87.9% and a specificity of 89.6% in the validation cohort (*n* = 620). Based on such improvements, a pan-cancer classifier using cfDNA methylation profiles has recently been applied to detect multiple types of malignancies and evaluate their tissue of origin; it achieved a positive diagnostic value in breast, colon/rectum, esophagus, head and neck, kidney, pancreas, prostate, and uterus, but encountered failure in lung cancer. Especially in early-stage lung cancer, its sensitivity was less than 50%.^9^ Another cfDNA methylation classifier for lung cancer named “PulmoSeek”^8^ enhanced the detection limit. However, its sensitivity (ranging from 0.610 to 1.000) and specificity (ranging from 0.200 to 0.875) cannot be optimized at the same time when setting different cutoff values. One possible reason for this issue was the high heterogeneity among different pathological types of lung cancer. Additionally, lung cancer undergoes less apoptosis and necrosis, thereby shedding fewer ctDNA fragments into the bloodstream. In this study, cfDNA methylation also achieved a promising diagnostic performance, with a sensitivity of 81% and a specificity of 59%. Moreover, the diagnostic model in this research includes only 9 markers, which could be detected using simpler techniques (such as methylation-specific PCR and gene chip), making its clinical application much easier. In addition, we also analyzed the biological functions of these markers based on previously published research. FIGNL2 promotes cell growth and tumorigenesis,^16^ while GFI1 shows methylation alteration^17^ in lung cancer. CHN1 is related to lymphoma and cervical carcinoma.^19^ PEAK3^20^ and myo7b^21^ promote cell proliferation, migration, and invasion in *in vitro* studies. TBCEL, UBE2S, and ZNF799 have been studied for their methylation changes and expression alterations, with established functions in multiple cancers.^18^ No research has been published on ZNF253. However, a larger sample size is needed to further enhance the diagnostic performance of this methylation model.

Most previous studies have demonstrated the concordance of DNA methylation among different biological samples.^35^^,^^37^^,^^38^ In this study, we also observed such a correlation between tissue and plasma in the paired patient subgroup. However, only a qualitative concordant relationship was evident when extended to the whole cohort (rho = 0.16). The extremely low percentage of ctDNA in cfDNA may be the most important reason. In patients with malignancy, less than 5% of DNA fragments in plasma originate from primary tumors, while approximately 80% are contributed by white blood cells.^29^^,^^39^ This may partly explain the modest correlation coefficient observed. Such inconsistency was also observed in Zhang's study.^35^ When comparing tissue samples with matched plasma samples, no significant correlations were found for the methylation alterations. Multiple methods have been applied to remove the interference of DNA fragments from leukocytes, including deconvolution analysis^36^^,^^40^ and cell-free methylated DNA immunoprecipitation and high-throughput sequencing (cfMeDIP-seq).^38^ However, their efficacies need further validation before clinical application.

Considering the advantage of methylation haplotype-based analysis in various malignancies,^27^^,^^29^^,^^35^ we tried this method using our sequencing data but did not achieve significant improvement. We divided the methylated haplotype regions according to Zhang's method. Only 20,000 of 147,888 methylated haplotype regions overlapped and were analyzed, while the remaining sites were omitted in the methylation haplotype-based analysis. Furthermore, Zhang identified the methylated haplotype regions in Caucasians, H1 human embryonic stem cells, and *in vitro* derived progenitors,^29^^,^^35^ while we used a Chinese population. More Chinese-based sequencing data is needed in future studies to identify a more accurate methylation haplotype system for cfDNA detection.

Our study also has several limitations in applying ctDNA as a diagnostic marker for early lung cancer. First, due to clinical practice issues, most of the tissue and plasma samples in our cohort were not collected from the same group of patients, limiting our ability to develop an integrated diagnostic model and evaluate its performance. Second, we used a capture-based DNA methylation assay that covers only approximately 200,000 CpG loci. To fully investigate the power and performance of ctDNA methylation in early lung cancer detection, especially in a liquid biopsy setting, it would be ideal to incorporate a larger number of CpG loci. Third, due to the low percentage of lung ctDNA signals in blood, our diagnostic model in plasma achieved an AUC of only 0.79. The current model needs improvement to be considered clinically practical. One possible improvement is to isolate exosomes and sequence only the exosomal DNA instead of directly measuring DNA methylation signals in the whole plasma. This method would generate a higher signal-to-noise dataset than the current approach. Another possible enhancement may be increasing the input of plasmic DNA since only 10 mL plasma was collected from each participant in this study. However, its clinical feasibility would be impacted when much blood was needed.

In the future, ctDNA methylation may have the potential to be an important biomarker for the diagnosis of early lung cancer. By integrating multi-modal information provided by computed tomography images, ctDNA mutations, and ctDNA methylation patterns, both the sensitivity and specificity of early lung cancer diagnosis could be dramatically improved. By collecting a larger dataset of tumor images and molecular profiles and developing novel computational methods, noninvasive approaches could become increasingly accurate and available for guiding clinical decisions.

In conclusion, this research demonstrated the feasibility of applying ctDNA methylation as a biomarker for lung cancer, although inconsistencies were observed in the plasma cohort. It may help to differentiate malignancy from benignity, avoid unnecessary invasive tests, and improve early diagnosis of lung cancer.

## Funding

This work was supported by the 10.13039/501100001809National Natural Science Foundation of China (No. 32201231).

## CRediT authorship contribution statement

**Lei Li:** Writing – review & editing, Writing – original draft, Resources, Data curation. **Kai Fu:** Visualization, Validation, Software, Formal analysis. **Xuyu Cai:** Data curation, Conceptualization. **Dan Liu:** Resources, Investigation. **Yingying Zhu:** Resources. **Weiwen Wang:** Validation. **Panwen Tian:** Investigation. **Ye Wang:** Validation. **Hui Xue:** Project administration, Conceptualization. **Michael P. Snyder:** Supervision, Project administration. **Weimin Li:** Writing – review & editing, Supervision, Project administration, Methodology, Funding acquisition, Data curation, Conceptualization.

## Conflict of interests

All authors declared no competing interests.

## Figures and Tables

**figs1:**
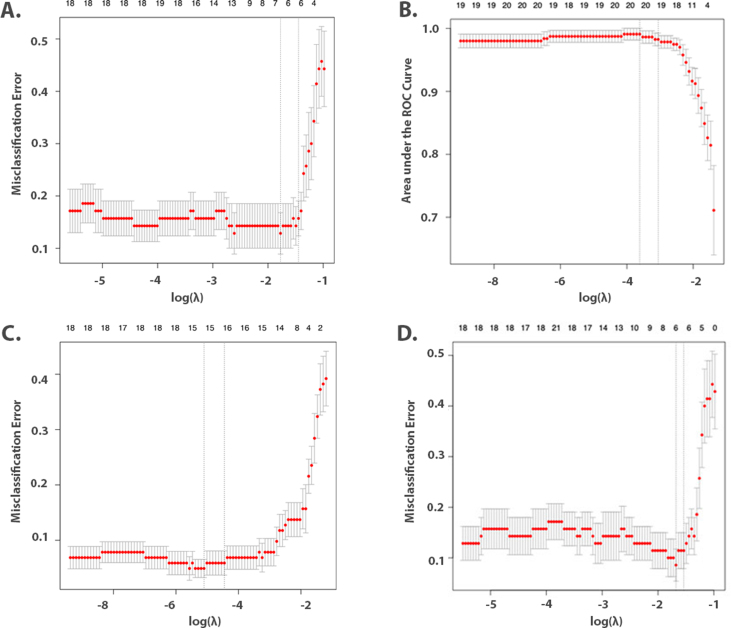


**figs2:**
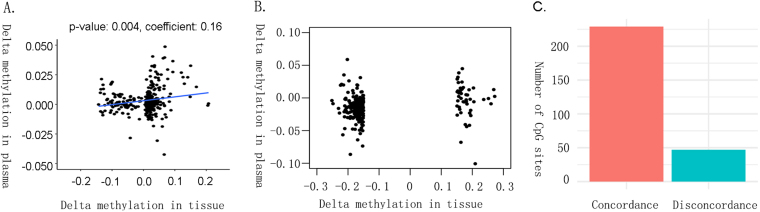


**figs3:**
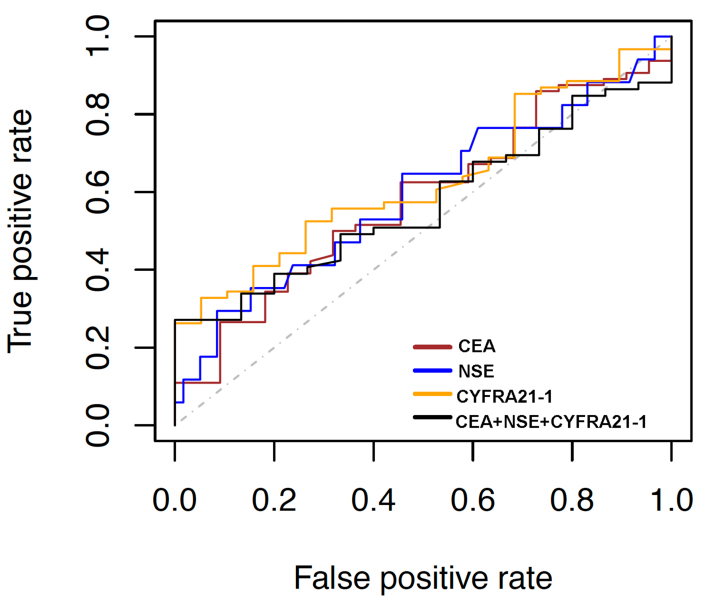


**figs4:**
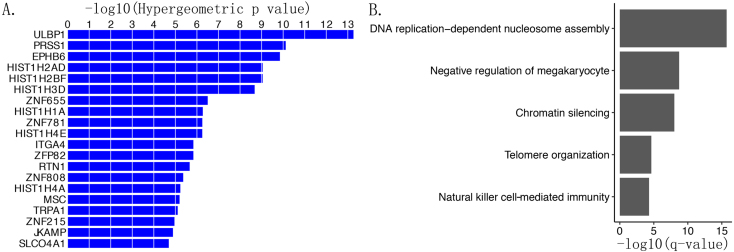


**figs5:**
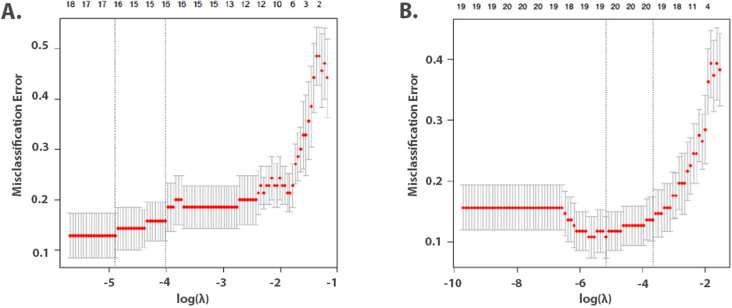


**figs6:**