# Supplementary material for: Diagnosis of early-stage non-small cell lung cancer using DNA methylation in tissue and plasma
Source: Genes Dis. 2025 Jan 28;12(6):101548. doi: 10.1016/j.gendis.2025.101548 (PMC12361987; doi:10.1016/j.gendis.2025.101548)
Supplement: Multimedia component 1 [file mmc1.docx]

**Supplementary Figures**

**Figure S1** Choosing lambda using the LASSO model for marker selection. **(A, B)** Choosing lambda in tissue cohort using the LASSO model for marker selection, in the training set (A) and the test set (B). **(C, D)** Choosing lambda in plasma cohort using the LASSO model for marker selection, in the training set (C) and the test set (D).

**Figure S2** Comparison of methylation alterations between tissue and plasma samples. **(A)** The correlation of methylation differences between benign/malignant samples in tissue and plasma in the paired patient cohort. **(B)** The correlation of methylation differences between benign/malignant samples in the tissue and plasma in the whole cohort. **(C)** The numbers of differential methylation sites in the concordance and discordance groups.

**Figure S3** Comparison of diagnostic performance among different cancer biomarkers clinically used at present.

**Figure S4** Methylation haplotype analysis in the tissue cohort. **(A)** Tissue DMR (differentially methylated region) enriched genes. **(B)** Gene ontology enrichment analysis.

**Figure S5** Choosing lambda using the LASSO model based on differentially methylated region (DMR). **(A)** Choosing lambda in tissue cohort based on DMR. **(B)** Choosing lambda in plasma cohort based on DMR.

**Figure S6** Diagnostic performances of differentially methylated region (DMR)-based models. **(A, B)** Receiver operator characteristic curve of the DMR-based diagnostic model for tissue cohort, in the training set (A)and test set (B). **(C, D)** Receiver operator characteristic curve of the DMR-based diagnostic model for plasma cohort, in the training set (C)and test set (D).
