# Supplementary material for: Diagnosis of early-stage non-small cell lung cancer using DNA methylation in tissue and plasma
Source: Genes Dis. 2025 Jan 28;12(6):101548. doi: 10.1016/j.gendis.2025.101548 (PMC12361987; doi:10.1016/j.gendis.2025.101548)
Supplement: Multimedia component 2 [file mmc2.docx]

**Supplementary Table 1. Characteristics and model weights of methylation markers in tissue samples**

| **Marker** | **Model Weight** | **Reference Gene** | **Annotation** | **Gene type** | **Description** |
| --- | --- | --- | --- | --- | --- |
| **7.25133699** | -1.451591699 | CYCS | Intergenic | Protein-coding | cytochrome c, somatic |
| **14.87511680** | -0.068729038 | LOC283585 | Intergenic | ncRNA | uncharacterized LOC283585 |
| **7.6703627** | 1.061195837 | ZNF316 | Intergenic | Protein-coding | zinc finger protein 316 |
| **19.32129899** | -1.51554355 | THEG5 | Intergenic | Protein-coding | testis highly expressed protein 5 |
| **15.34787100** | 2.555949458 | MIR1233-1 | Intergenic | ncRNA | microRNA 1233-1 |
| **12.54834099** | -2.398057257 | LOC102724050 | intron | ncRNA | uncharacterized LOC102724050 |
