# Supplementary material for: Diagnosis of early-stage non-small cell lung cancer using DNA methylation in tissue and plasma
Source: Genes Dis. 2025 Jan 28;12(6):101548. doi: 10.1016/j.gendis.2025.101548 (PMC12361987; doi:10.1016/j.gendis.2025.101548)
Supplement: Multimedia component 3 [file mmc3.docx]

**Supplementary Table 2. Characteristics and model weights of methylation markers in plasma samples**

| **Marker** | **Model Weight** | **Reference Gene** | **Annotation** | **Gene type** | **Description** |
| --- | --- | --- | --- | --- | --- |
| **11.12089474** | -17.45606011 | TBCEL | Promoter-TSS | protein-coding | tubulin folding cofactor E-like |
| **19.55915707** | -0.839017891 | UBE2S | Exon | protein-coding | ubiquitin-conjugating enzyme E2S |
| **2.128324253** | -6.351425252 | MYO7B | Exon | protein-coding | myosin VIIB |
| **1.92949802** | -0.745036136 | GFI1 | Promoter-TSS | protein-coding | growth factor independent 1 transcription repressor |
| **19.12524811** | 7.556002382 | ZNF799 | Intron | protein-coding | zinc finger protein 799 |
| **12.52241887** | -0.125819021 | FIGNL2 | Intron | protein-coding | fidgetin like 2 |
| **19.19971766** | 4.485329611 | ZNF253 | Promoter-TSS | protein-coding | zinc finger protein 253 |
| **19.2273801** | -13.90996354 | PEAK3 | Promoter-TSS | protein-coding | PEAK family member 3 |
| **2.175869998** | -17.71412805 | CHN1 | Exon | protein-coding | chimerin 1 |
